# Spontaneous Rupture of a Renal Artery Branch Aneurysm in a Young Man: A Rare Case of Retroperitoneal Hemorrhage

**DOI:** 10.7759/cureus.88369

**Published:** 2025-07-20

**Authors:** Bourhan Alrayes, Mohammad F Albattah, Ali Abouseni, Bishr Shibani, Monther Wahoud Al Kassab

**Affiliations:** 1 General Surgery, Islamic Hospital, Amman, JOR; 2 Orthopaedic Surgery, Islamic Hospital, Amman, JOR; 3 Science and Technology, Nottingham Trent University, Nottingham, GBR; 4 Trauma and Orthopaedics, Colchester Hospital, Colchester, GBR

**Keywords:** branch aneurysm, coil embolization, renal artery aneurysm, retroperitoneal hematoma, spontaneous rupture, wunderlich syndrome

## Abstract

Renal artery aneurysms (RAAs) are rare vascular abnormalities, with branch aneurysms being even more uncommon. Spontaneous rupture of a branch RAA without prior trauma, infection, or known risk factors is extremely rare and can be life-threatening. We report a case of a previously healthy 22-year-old male patient who presented with sudden left flank pain and hematuria. Initial imaging revealed a large retroperitoneal hematoma; however, the source of bleeding was not immediately apparent. Following conservative management, a delayed contrast-enhanced CT and angiography identified a ruptured left renal artery branch aneurysm. The patient was successfully treated with coil embolization and recovered uneventfully. This case highlights the importance of considering RAA rupture in young patients with spontaneous retroperitoneal hemorrhage, supports the role of conservative management with close observation, and the role of angiographic embolization in cases of unclear bleeding sources.

## Introduction

Renal artery aneurysms (RAAs) are rare vascular abnormalities, first described centuries ago, with modern autopsy and angiographic series estimating their prevalence at approximately 0.1% of the population [1,2]. Early clinical observations, such as those reported by Poutasse, highlighted the rarity and serious complications associated with RAAs [1]. Further studies demonstrated that most RAAs remain asymptomatic and are often discovered incidentally during imaging performed for unrelated reasons [2-4]. Commonly associated risk factors include fibromuscular dysplasia, atherosclerosis, connective tissue disorders, and inflammatory vasculopathies [4-6].

While many RAAs are clinically silent, rupture represents a critical and potentially fatal event. Historical series reported mortality rates approaching 80% following rupture [1,3]. Although advances in diagnostic imaging and endovascular therapies have improved outcomes, the risk of rupture persists, particularly in larger aneurysms or during pregnancy [2,7,8]. Even in non-pregnant patients, spontaneous rupture, although rare, can result in life-threatening retroperitoneal hemorrhage requiring urgent intervention [8].

Branch RAAs, involving segmental or intrarenal arteries, are even less common than main renal artery aneurysms [5,6]. Their clinical presentation is often nonspecific, including flank pain, hematuria, or hemodynamic instability, which can mimic more common conditions such as urolithiasis or renal trauma [9-12]. Consequently, diagnosis may be delayed, particularly in young, otherwise healthy patients with no identifiable risk factors. Several case reports have emphasized the challenges in identifying ruptured branch RAAs, underlining the necessity for heightened clinical suspicion [11,12].

Accurate diagnosis relies mainly on imaging. Non-contrast CT typically reveals retroperitoneal hematoma but may fail to identify the bleeding source [13,14]. Contrast-enhanced CT angiography (CTA) improves detection by delineating vascular structures and active extravasation [9,13]. However, digital subtraction angiography (DSA) remains the gold standard for definitive diagnosis and allows for immediate endovascular intervention when necessary [8,10]. Point-of-care ultrasound has also been proposed as a valuable adjunct in emergency settings for early detection [9].

Treatment approaches have evolved substantially over the past decades. Historically, open surgical repair, including aneurysmectomy and arterial reconstruction, was the treatment of choice [1,4]. However, with the advent of minimally invasive techniques, endovascular approaches such as coil embolization and covered stent placement have become preferred in suitable patients, offering high technical success rates and reduced morbidity [6,8,10]. Selective angiographic interventions, e.g., embolization, are advantageous for branch aneurysms, allowing for effective hemostasis while preserving renal parenchyma [8,10].

## Case presentation

A 22-year-old male smoker with recently diagnosed hypertension and psoriasis presented to the emergency department with a four-day history of left flank pain radiating to the left upper quadrant and groin. The pain was moderate (6 out of 10) and associated with sudden dizziness, weakness, blurring of vision, one episode of vomiting, and gross hematuria. The review of other systems was negative. He was taking carvedilol for hypertension and topical betamethasone for psoriasis. His family and surgical histories were unremarkable. 

**Figure 1:**
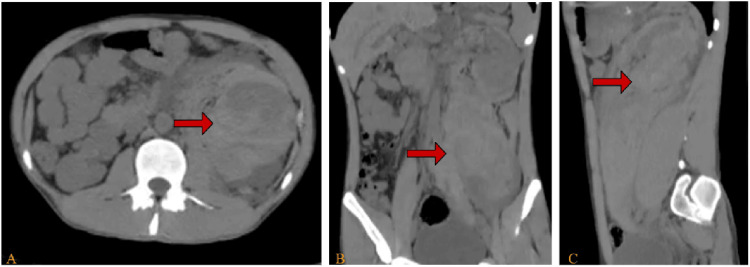
Axial (A), coronal (B) and sagittal (C) views of abdomen and pelvis CT without contrast at admission showing a huge left perinephric retroperitoneal hematoma measuring 20 x 12 x 4.5 cm

On examination, vital signs were normal except for elevated blood pressure. Abdominal examination revealed mild left abdominal and left costovertebral angle tenderness. Laboratory investigations showed elevated C-reactive protein (49 mg/dl) and serum creatinine (1.46 mg/dl). An abdominal CT scan without IV contrast revealed a large perinephric and retroperitoneal hematoma measuring about 20 x 12 x 4.5 cm, causing a mass effect on the spleen and adjacent bowel (Figure 1).

The patient was admitted to the ICU for conservative management with nil per os (nil by mouth), IV fluids, blood preparation, and urine output monitoring. Isosorbide dinitrate infusion was initiated for high blood pressure control, and two units of packed red blood cells were administered as recommended by the urology team due to persistent hemoglobin decline (Table 1).

**Table 1:** Laboratory results over time, including transfusion events PRBC: packed red blood cells; PT: prothrombin time; aPTT: activated partial thromboplastin time; INR: international normalized ratio; WBC: white blood cells; RBS: random blood sugar "-" indicates test not performed; * a blood transfusion was given shortly before this result was obtained

Day	Time	Hemoglobin (g/dL)	WBC (×10³/μL)	Platelets (×10³/μL)	Urea (mg/dL)	Creatinine (mg/dL)	PT	aPTT	INR	Random Blood sugar	Notes
Day 1	04:00	12.6	13.9	503	28	1.48	17.6	29	1.35	162.9	
	11:41	10.6	-	-	-	-	-	-	-	-	1 st transfusion (2 units of PRBC)
	19:36	8.6*	-	-	-	-	-	-	-	-	
Day 2	06:24	9.8	-	-	-	1.15	-	-	-	-	
	16:14	8.7	-	-	-	-	-	-	-	-	
Day 3	06:24	10.9	-	-	-	0.93	-	-	-	-	2nd transfusion (1 units of PRBC)
	12:52	8.3*	-	-	-	-	-	-	-	-	
	19:08	10.9	-	-	-	-	-	-	-	-	
Day 4	06:32	11.0	-	-	17.1	0.97	-	-	-	-	
Day 5	07:25	11.5	-	-	-	-	-	-	-	-	
Day 6	06:11	11.5	-	-	-	1.07	15	32.9	1.14	-	
Day 7	06:31	11.5	-	-	-	0.97	-	-	-	-	
Day 8	07:39	11.0	-	-	-	0.95	-	-	-	-	

**Figure 2:**
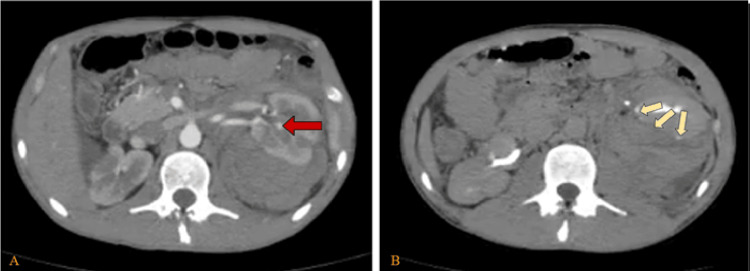
Abdomen and pelvis CT with IV contrast on day 2 (A) Arterial phase showing a huge left perinephric retroperitoneal hematoma with no change in its size with little arterial blush (red arrow). (B) Delayed phase showing a rim of contrast extravasation around the left kidney (yellow arrows) with suspected cortical defect reaching its pelvic seen in the midportion, an hypoenhancing area in the upper pole, suggesting urinary extravasation

On the second day, renal function improved, and oral intake was resumed. Thus, contrast-enhanced CT showed a stable hematoma with a suspected small contrast blush at the mid-pole and delayed rim enhancement suggesting urinary extravasation (Figure 2). Conservative management continued.

**Figure 3:**
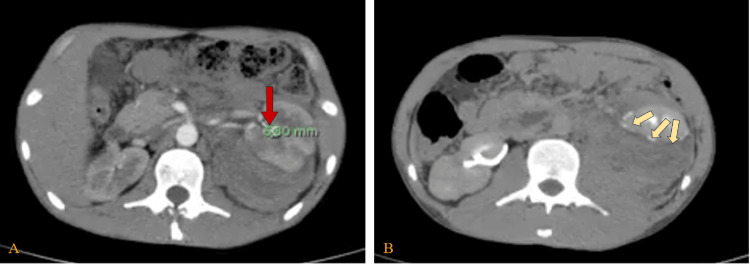
Repeat abdomen and pelvic CT with IV contrast on day 5 (axial view) (A) Arterial phase showing haematoma decreased in size slightly and increase in the size of contrast blush at the mid pole posteromedially represents an active bleeding (green mark and red arrow). (B) Delayed phase showing a rim of contrast extravasation around the left kidney (yellow arrows) with suspected cortical defect reaching its pelvic seen in the midportion and hypoenhancing area in the upper pole, suggesting urinary extravasation

On the third day, hemoglobin dropped to 8.9 g/dL, prompting another transfusion of one packed red blood cell. Vital signs remained stable. Further evaluation for secondary hypertension was unremarkable. Hypertension was treated with oral medication with partial response, as it was linked to the new left retroperitoneal hematoma. Despite improved renal function, persistent hypertension and subtle imaging changes prompted a repeat CT on day five, which showed increased size of the contrast blush, suggestive of active bleeding (Figure 3).

**Figure 4:**
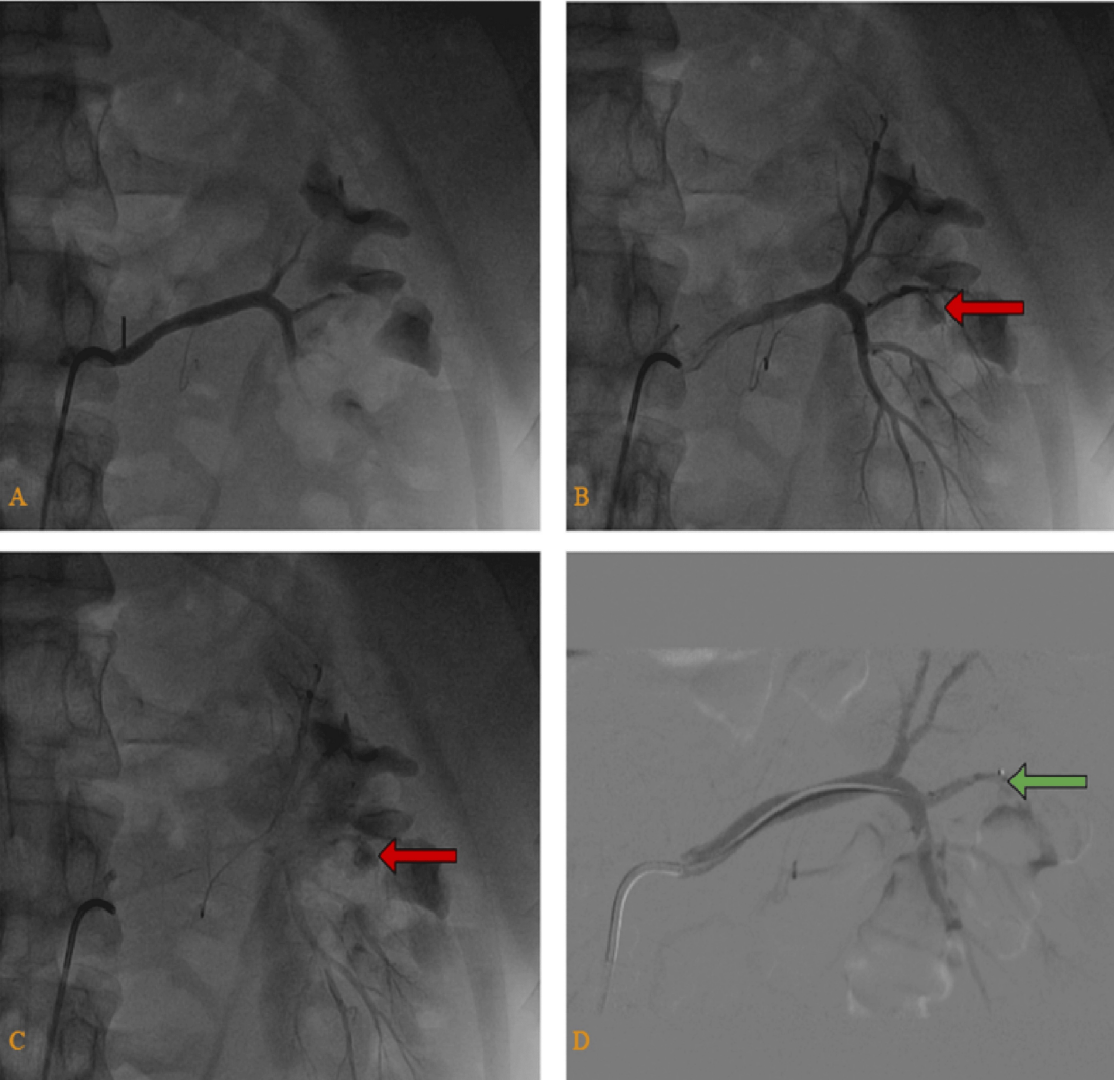
Angiography of left renal artery (A) Initial phase contrast did not reach the aneurysm. (B) Contrast reaches the aneurysm and extravasation of contrast is seen (red arrows). (C) Washout phase; partial extravasation is still seen (red arrows). (D) Embolization done for the aneurysm (green arrow).

**Figure 5:**
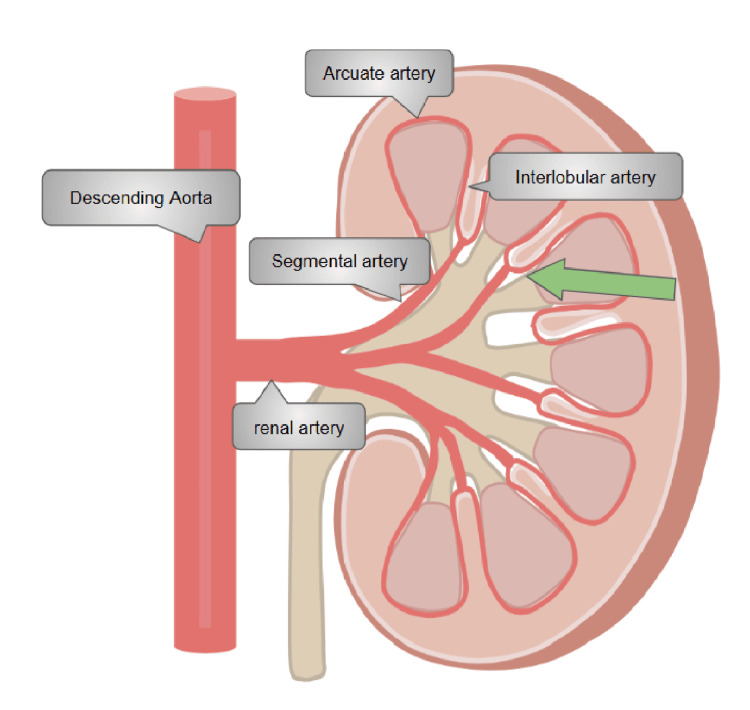
Anatomical division of renal artery The illustration shows the anatomical functional branches of the left renal artery, indicating the descending aorta, main renal artery, segmental artery, and interlobular artery. The aneurysm was in the intralobular artery of the middle segmental branch (green arrow). Image Credit: Bourhan Alrayes, Author

Interventional radiology was consulted, and angiography revealed active bleeding from a left renal artery branch aneurysm. Coil embolization was performed successfully (Figure 4). Figure 5 shows an illustration of the branching of the renal artery and the site of the aneurysm. 

The patient reported significant relief of pain, tolerated the diet, and was discharged the next day on oral antibiotics and oral antihypertensive medication. At the one-month and six-month follow-up visits, the patient was doing well. 

## Discussion

RAAs refer to localized dilation of the renal artery or its branches. Initially recognized as the first renal artery pathology, they were historically perceived as uncommon (about 1% of the population) until the advent of widespread angiography [1-4]. Historically, the first documented case of a ruptured RAA was reported in 1770 [15]. There are four structural types of RAAs: saccular, fusiform, dissecting, and arteriovenous/microaneurysms [2]. Saccular aneurysm is the most common form and constitutes approximately 80% of RAAs [5]. Spontaneous rupture of an aneurysm is rare. Risk factors for the rupture of an aneurysm include size > 2 cm, hypertension, and pregnancy [7]. 

RRA is usually asymptomatic [8,9]. However, up to 20% of patients could present with abdominal and/or flank pain, hematuria [1,5]. The average age for presentation is the sixth decade of life, with female predominance [5]. CT is currently the most frequently used diagnostic method, followed by MRI, ultrasonography, and catheter-based arteriography [6]. Rupture of RAA can cause severe deterioration of the patient's condition, leading to urgent nephrectomy [11]. However, there are other reported cases of RAA rupture causing retroperitoneal hematoma, which were treated conservatively without intervention [12]. Moreover, rupture of the RAA could contribute to a minority of causes of spontaneous retroperitoneal hematoma and present as vague abdominal pain [13], as in our patient. 

Wunderlich syndrome (WS) is defined as a spontaneous renal capsule apoplexy, resulting in subcapsular or perirenal space hemorrhage in patients with no prior trauma [16,17]. It is named after Carl Wunderlich, who first described a patient with spontaneous subcapsular and perinephric bleeding without preceding trauma [18]. In 85% of cases, WS has been associated with renal tumors; however, ruptured RAAs, including both true aneurysms and pseudoaneurysms, are a rare cause of WS [19,20]. 

In one published series of 13 patients, only two patients demonstrated the classic Lenk's triad of pain, mass, and hypovolemic shock, while all 13 patients had flank pain [21]. This is similar to our case, in which the patient exhibited only one component of Lenk’s triad. 

The management of RAAs is guided by the patient’s overall clinical stability and the anatomical location of the aneurysm. Further decline in hemoglobin levels is a recognized indication for interventional management of ruptured RAA [5]. Traditionally, open surgical repair, such as aneurysmectomy with arterial reconstruction, was considered the standard treatment [1,4]. However, the emergence of minimally invasive techniques has shifted the preferred approach toward endovascular interventions, including coil embolization and covered stent placement, which have demonstrated high technical success rates with lower associated morbidity [6,8,10]. Selective embolization, in particular, offers significant advantages in the treatment of branch aneurysms by achieving effective hemostasis while preserving renal parenchymal function [8,10].

## Conclusions

Spontaneous rupture of a renal artery branch aneurysm is a rare but potentially fatal cause of retroperitoneal hemorrhage. Early diagnosis is challenging due to subtle initial imaging findings. This case underscores the importance of delayed angiography in persistent or unexplained retroperitoneal bleeding and supports endovascular coil embolization as an effective, kidney-preserving treatment.
